# Tracking down the Candy Crush Terrorist: the fragile relation between gaming motives and radical attitudes

**DOI:** 10.3389/fpsyg.2025.1585576

**Published:** 2025-10-14

**Authors:** Simon Greipl, Maximilian Lechner, Jannik Fischer, Heidi Schulze, Julian Hohner, Diana Rieger

**Affiliations:** ^1^Department of Media and Communication, LMU Munich, Munich, Germany; ^2^Faculty of Law, University of Hamburg, Hamburg, Germany

**Keywords:** gaming, motivation, radicalization, extremism, structural equation modeling, latent class analysis

## Abstract

The gaming ecosystem is increasingly observed with the concern that it could pose a threat to public safety, and research accumulates evidence for blatant extremism in the surrounding online space of games. Currently, a connection between gaming and extremism can be established through identity related processes, e.g., gaming-related radicalization elements, distal to gaming itself, such as gaming communities and culture. However, this also raises the question of what the precise function of proximal gaming factors, such as gameplay, mechanics, stories, or game-play motivations, is in the relationship between gaming and extremism. This article aims to shed light on the relation of gaming and extremism by identifying individual profiles of videogame playing based on gameplay motivations and linking them to indications of radical attitudes (here: xenophobia and violence acceptance) as well as conspiracy beliefs that can be associated with extremist beliefs. Further, we include marginalization and anomie as mediators to gain comparative and fine-grained information about the sole impact of gaming motives on radical attitudes. Our findings indicate that while few motivational profiles exhibit weak yet direct connections to radical attitudes, others display the opposite pattern, suggesting a more complex relationship. Marginalization and anomie strongly predict most radical outcome variables and mediate the relationship in most cases, however sometimes negatively. We only found one complex motivational profile that substantially leans toward late-stage radical attitudes, while for instance, dominant social motives clearly inhibit radical outcomes. The current study thus deflates any straightforward perspective on the becoming of a ‘radical gamer'.

## 1 Introduction

The association between terrorist attackers and gaming environments continues to fuel debates about the role of gaming culture and communities in the emergence of delinquency, radicalization, and extremism.^1^ For instance, the Christchurch and Halle terror attacks were live-streamed and used gamification strategies that mimicked first-person shooter games (Lakhani and Wiedlitzka, 2023; Evans, 2019). Given the sheer ubiquity of gaming—over 3 billion people around the planet play video games (DFC Intelligence, 2020)—public discourse often fluctuates between alarmist perspectives—painting gaming as a dystopian breeding ground for extremism—and defensive reactions from gaming communities that reject such characterizations outright. Empirical research on the potential link between (violent) video games and real-world violent behavior has produced, at best, mixed results, emphasizing that any relationship between gaming and extremism is, if it exists, complex rather than straightforward. This suggests that the digital habitat—often broadly labeled as “gaming” or “gaming communities”—is still misunderstood and generalized and requires a more nuanced approach.

Even if the current state of literature on the topic is rather selective, anecdotal, or theoretical, and equating gaming with extremism would be premature, overlaps between gaming environments and radicalization processes seem to exist (Ebner, 2019; Vaux et al., 2021). For instance, the EU Terrorism Situation and Trend Report explicitly notes that the age of individuals involved in far-right online communities continues to decrease, likely influenced by increased online activity during the COVID-19 pandemic. The report further highlights that gaming platforms are increasingly being exploited by right-wing extremists to spread propaganda, often through gamified fascist narratives (Europol, 2022). While research has started to focus on the surrounding digital ecosystem of gaming, unanswered questions more narrowly tied to gaming itself remain, such as: how and what aspects of gaming-related behaviors, such as gaming habits and motivations, can shape pathways toward radicalization? In this paper, we specifically explore gaming motivations as starting point for a more nuanced stance on the relation between gaming and extremism. “Traditional” research on games and gaming has emphasized the role of playing motivations in relation to *problematic or excessive gaming behaviors*, sometimes discussed under the umbrella of gaming addiction (Kneer and Rieger, 2015; Kneer et al., 2014). The present study extends previous research that has identified gaming motivations as significant predictors of various maladaptive and harmful outcomes, exploring their potential role in adapting extremist attitudes.

## 2 Gaming as an online ecosystem exploited by extremists

The sheer ubiquity of gaming seems insufficient in explaining why it is so easy to come across blatant extremism on some gaming platforms (Vaux et al., 2021; Wells et al., 2024; Anti-Defamation League, 2020). One key factor may lie in the structural affordances of the gaming sphere that make it particularly advantageous for extremist actors.

First, gaming communication spaces are, *decentralized, private*, and *less moderated* compared to mainstream platforms like Facebook or X. Relevant communication in gaming is distributed across a wide range of platforms, from in-game communication (e.g., text/voice chat directly “in-game” or via Teamspeak) to those primarily attached to gaming (e.g., Steam, Discord, Twitch) to conventional and unrelated platforms (e.g., Youtube, Twitter). Moderation efforts by platform companies often push extremists into alternative and private digital spaces (e.g., Trovo) (Davey, 2021). In some cases, these platforms may even serve as functional substitutes for traditional social media in extremist networking, making gaming platforms an appealing infrastructure for ideological dissemination and recruitment (EU, 2020).

Second, the reach of gaming as a youth-oriented mass pop cultural phenomenon makes it interesting for extremist *recruitment*. Especially jihadists used video games to tap into the pop culture of young people (Lakomy, 2019). The recruitment potential of gaming is often discussed (Ostovar, 2017; Weimann, 2015; Lakomy, 2019), yet little is known about its actual extent.

Third and most importantly, gaming communities can be a place in which like-minded individuals meet, and ideologies can resonate uninhibitedly (Mølmen and Ravndal, 2021). Ongoing (positive) engagement with people who share radical or extreme worldviews can strengthen radical ideas and normalize behaviors that typically deviate from democratic norms (Neumann, 2013). Online fringe groups often provide a gathering space for individuals who feel disillusioned, angry, or fearful of political institutions, potentially fostering a radicalization-friendly environment (Urman and Katz, 2022). As a result, gaming spaces can offer extremists both a communication hub and an ideological incubator.

Current literature challenges the assumption that recruitment is among the primary intentions of extremists' activities in online gaming communities. Instead, by offering attachment and identity (Kowert and Oldmeadow, 2015), it is hypothesized that reinforcing existing beliefs may be the more relevant, yet neglected strength of gaming (communities) (Robinson and Whittaker, 2021; Vaux et al., 2021). Certain gaming cultures can facilitate the development of fused identities, in which individuals strongly align with the collective identity of a group (Kowert et al., 2022). When this occurs, individuals may be more likely to internalize socially harmful attitudes (e.g., racism, sexism) and, in extreme cases, express willingness to defend their in-group through violence. Moreover, research suggests that gamers with highly fused identities are particularly susceptible to adopting anti-social attitudes and behaviors when embedded in toxic rather than non-toxic gaming communities (e.g., Call of Duty vs. Minecraft; Kowert et al., 2022). This distinction underscores both the generalized and specific radicalization potential present within certain segments of gaming and its communities (White and Lamphere-Englund, 2024), but also the complexity that results in inconsistent reporting of the issue (Collison-Randall et al., 2024).

## 3 Proximal and distal gaming factors and their association with extremism

**Figure 1:**
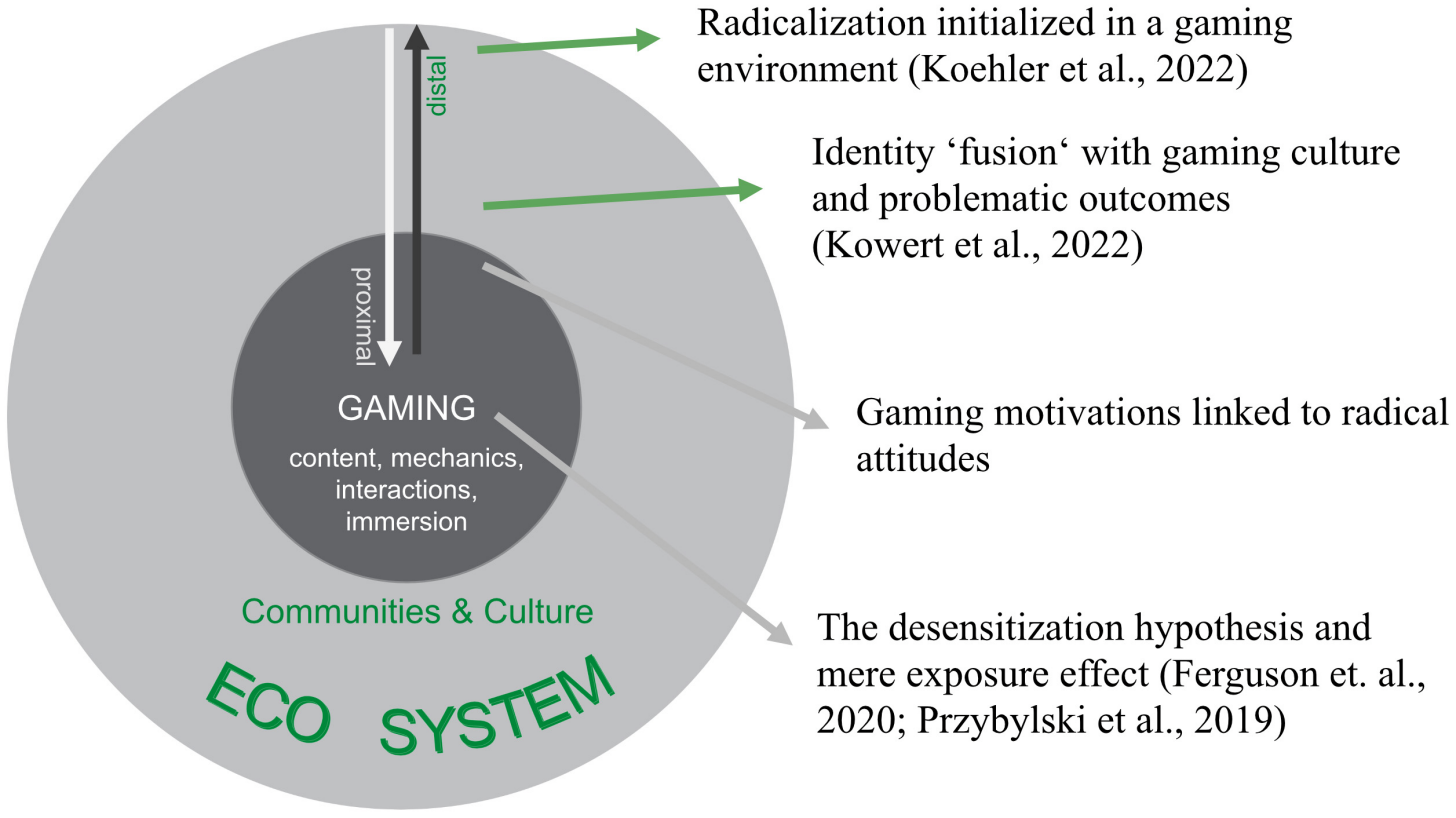
Positioning of prior research and the current study along a continuum of distal (e.g., online communities, platforms) to proximal (e.g., gaming motivations) gaming-related radicalization factors or the nexus between gaming and extremism. Rectangles represent conceptual areas discussed in the literature.

Understanding the potential link between gaming and extremism requires disentangling the different factors that may contribute to radicalization processes. A distinction can be made between *distal* gaming-related factors, which are external to gameplay but embedded in gaming culture and communities (Ferguson et al., 2020; Przybylski and Weinstein, 2019). These factors typically involve identification processes and the ways in which communities are formed and maintained, including, for instance, the use of specific language codes within a community. Further, there are *proximal* gaming-related factors, which relate directly to the mechanics, narratives, and motivational structures of gaming itself (see Figure 1).

Distal factors do not inherently arise from gaming as an activity but may shape radicalization processes through the broader gaming ecosystem. Any potential for radicalization requires a conducive environment, which can be understood through the 3N Theory of Radicalization (Kruglanski et al., 2019), consisting of needs, narratives, and networks. First, radicalization is often driven by the frustration of existential needs, such as the desire for recognition, value, and respect—needs that can be violated by experiences of deprivation, discrimination, exclusion, or victimization (McCauley and Moskalenko, 2008; Ngari and Reva, 2017; Pfundmair et al., 2022). However, frustrated needs alone are rarely sufficient to initiate radicalization (Kruglanski et al., 2019). Radicalization processes are further shaped by opportunity factors, including narratives, which provide ideological justification for radicalization (Moghaddam, 2005), and networks, which enable collective radical action (Sageman, 2008). The gaming ecosystem and its communities can facilitate all three of these prerequisites. Radicalization within gaming spaces is, therefore, not simply a byproduct of gaming itself but emerges from the interplay of personal vulnerabilities and gaming-related social contexts (Kowert et al., 2022; Fizek and Dippel, 2020).

Beyond these external influences, proximal gaming-related factors may also play a role in facilitating pathways to radicalization. These encompass elements directly tied to gameplay and game design, including players' motivations for gaming and the reinforcement of ideological narratives within games themselves. Recent research suggests that understanding gaming's role in extremism requires moving beyond a focus on visual representations of ideology (e.g., depictions of enemies, in-game iconography) and instead examining how ideological messages are embedded in the interactive and procedural rhetoric of gameplay (Robinson and Whittaker, 2021). Extremist narratives, such as nostalgic glorifications of an idealized past, are commonly found in both radical ideologies and certain video game storylines (Hong, 2015).

Interactive and immersive game environments allow players to experience these ideological narratives firsthand, which may reinforce motivations to return to an illusionary “golden age” (Schlegel, 2020). Similar mechanisms have been proposed for heroic storytelling (Kuss, 2013), particularly in games that emphasize hypermasculine portrayals of power and dominance, a feature that has long been criticized in gaming culture (Condis, 2018). Customization of avatars can heighten identity immersion, and in turn amplify the aggressive impact of violent content (Fischer et al., 2010). Other game mechanics—such as rewarding violent behavior while minimizing its consequences—have been hypothesized to increase susceptibility to radicalization by normalizing violent action and strengthening players' sense of efficacy through mastery experiences (Schlegel, 2020).

Proximal gaming-related factors can also be mapped onto Kruglanski's 3N Theory. First, gaming inherently serves fundamental psychological needs, such as autonomy, competence, and relatedness, as outlined in Self-Determination Theory (Ryan et al., 2006; Przybylski et al., 2010). This framework has been extensively used in game studies (Wohn et al., 2020; Rieger et al., 2014) to explain gaming's strong motivational pull. The taxonomy of gaming motivations (Yee, 2006) distinguishes between achievement, social interaction, and immersion-based motives, each of which has been linked to distinct behavioral and attitudinal outcomes. For instance, escapism-driven gaming motives (a component of immersion) have been associated with problematic gaming behaviors (Kneer and Glock, 2013), while social gaming motives have been linked to positive effects, such as enhanced wellbeing (Bowman et al., 2022).

The other two pillars of Kruglanski's theory—narratives and networks—are opportunity factors that are directly embedded in many games. Core ideological narratives are often embedded into game storylines alongside competitive and cooperative multiplayer game formats. This way, e.g., by shaping social dynamics and group identities by multiplayer formats, proximal and distal factors are usually interwoven. To explore the intersection of gaming and extremism and contributing to the systematization of research on this nexus, the present study focuses specifically on need-related proximal gaming-related factors by examining how motivational profiles for playing video games relate to radical attitudes. These are analyzed in comparison to established predictors of radicalization, such as experiences of marginalization and anomie (Hayes, 2017; Lyons-Padilla et al., 2015).

## 4 Motivational profiles as susceptibility factors for radicalization?

To the best of our knowledge, no study has yet examined proximal game-related factors, such as the direct effects of gameplay and gaming motivations, in relation to radicalization. Gaming motivations vary significantly among players, similar to how user types respond differently to gamified features (Chou, 2016). These motivational patterns may be a useful proxy to gaming-related individual vulnerabilities. Preliminary theoretical works in the field of gamification have begun to map player types with respect to their potential susceptibility to radicalization processes (Schlegel, 2021). We extend this work by building on an established motivational typology in video game research and enrich the theoretical outlook on radicalization potentials with evidence from related fields, such as toxic player behavior, gaming addiction, negative psychosocial outcomes, and other problematic or excessive gaming behaviors (Kneer and Glock, 2013; Cheah et al., 2022; Wang et al., 2021). For the purposes of this study, we classify player motivations based on Yee's (2006) typology, condensing them into four central player types: the socializer, the escapist (e.g., gaming as a coping mechanism), the competitor (e.g., achievement-oriented gaming), and an added profile, the hedonist (driven primarily by entertainment and enjoyment).

### 4.1 The socializer

Socializers play video games primarily to build and maintain social relationships (Kahn et al., 2015). They tend to have greater in-game social capital (Kahn et al., 2015; Williams, 2006), and social aspects of gaming are particularly important for many players (Rapp, 2017). Social interactions in gaming spaces can significantly shape player behavior (Evans et al., 2021; Barr and Copeland-Stewart, 2022), and social motives for video gaming are considered beneficial for the physical and mental health of players (Billieux et al., 2013; Bowman et al., 2022). Therefore, the socializer does not appear to be associated with any tradition linked to problematic behavioral or psycho-social outcomes (Wang et al., 2021).

However, social connection is central to any extremist action (Horgan, 2008). Social attachment can induce a state of “readiness” for radicalization (Al Raffie, 2013) and drives young people to join an extremist group (Venhaus, 2010). Individuals with insecure identities may be particularly vulnerable to highly entitative groups, such as extremist communities, which offer a sense of belonging and ideological cohesion (Hogg and Adelman, 2013). Additionally, gaming for social status and recognition, such as developing and maintain self-esteem and life satisfaction as well as identity, can be indicative for questionable video gaming (Delfabbro and King, 2015).

### 4.2 The escapist

Escapism is associated with low self-esteem, greater negative affect, and a tendency toward impulsive behavior (Billieux et al., 2015). Compared to achievement-oriented players (e.g., competitors), escapists tend to exhibit higher levels of stress and depression (Ballabio et al., 2017). Thus, escapist players use video games to distract themselves from real-life problems (Kahn et al., 2015), more specifically to get away from discomfort, boredom, and negative emotions (Laor, 2020).

Extensive research has linked escapist motives in gaming to negative psychological and social outcomes (Kirby et al., 2014). Several studies suggest that escapism negatively impacts psychological wellbeing (Goh et al., 2019; Griffiths, 2010). (Caplan et al. 2009) found escapism to be one of the strongest predictors of problematic internet use and online gaming. This is particularly concerning when escapism serves as a coping strategy for real-life difficulties, as it has been associated with social withdrawal and isolation (Stetina et al., 2011). During the COVID-19 lockdowns, for instance, gaming was frequently discussed as a means to escape feelings of isolation (Zhu, 2021).

Extremist groups and ideologies may serve a similar function, providing individuals with an alternative identity and sense of purpose to evade real-world problems—a dynamic comparable to substance abuse as an escape mechanism (Al-Attar, 2019). As such, escapists may be particularly susceptible to conspiracies or even extremist narratives, as they offer simple solutions to complex problems (Douglas et al., 2019). Excessive gaming shows a weak link to aggression (Grüsser et al., 2007) which could be mediated by escapism. However, recent studies suggest that escapism can also lead to positive psychological outcomes (Stenseng et al., 2021).

### 4.3 The competitor

Competition is considered a component of achievement and implies a propensity to challenge and challenge others, climb rankings, and establish dominance (Yee, 2006), from which competitors draw their enjoyment and intention to continue the gameplay (Lin et al., 2012). It increases their ardor and thereby fosters their craving for achievement (Fuster et al., 2014) where overcoming challenges creates a sense of mastery, fulfilling the need for competence (Wulf et al., 2019). Many competitive multiplayer games, particularly those in the shooter genre, incorporate direct competition as a fundamental mechanic.

Highly competitive gaming environments have been associated with toxic masculinity (e.g., Maloney et al., 2019)—a concept referring to hypermasculine ideals of dominance, aggression, and emotional restraint that alludes to societal standards for boys and men that imply anger, callousness, and hostility as appropriate social behaviors (Blackburn and Scharrer, 2019). These norms have been linked to debatable attitudes toward gender and social interactions, including hostility and acceptance of sexual harassment (Levant and Richmond, 2007).

Ambitious competitors seek to win to climb the rankings (Robson et al., 2016). But their pursue of prestige and social status (Sailer et al., 2017) can, at some point, take on maladaptive forms in terms of toxic behavior (Kou, 2020). Some research suggests that highly competitive individuals may be particularly sensitive to external validation and status hierarchies and may be more dependent on external rewards, which could contribute to susceptibility to extremist ideologies that emphasize dominance and social order (Schlegel, 2021).

### 4.4 The hedonist

A significant body of research has identified a strong relationship between enjoyment, engagement, and wellbeing in gaming (Rieger et al., 2014). Enjoyment in gaming is largely derived from the satisfaction of autonomy, competence, and relatedness—the three fundamental psychological needs outlined in Self-Determination Theory (Ryan et al., 2006). Players with a hedonic motivation engage in gaming primarily for entertainment, and their gaming motives do not inherently suggest a predisposition toward radicalization. A dominant hedonism or entertainment motive might also be present in so called casual gamers. These gamer types are usually less tied to online gaming communities and rather infrequent gamers, therefore they do not show an increased risk of susceptibility to extremist ideologies.

### 4.5 Complex and extreme motives

While novice gamers may simply seek positive rewards (Brand et al., 2019), many gamers—especially more experienced ones—develop more complex motivational profiles (Cheah et al., 2022). How complex motives may interact in a way that facilitates or inhibits a susceptibility to extremism is currently an exploratory field of this study. Strong motivation linked to gaming or a gaming community, for instance, can lead to increased readiness to use violence on behalf of the group (Kowert et al., 2022). Another phenomenon is that fun and emotional arousal is pursued at the expense of others, leading to toxic or harmful in-game behaviors (Schmitt, 2017). In these contexts, disruptive or harmful behavior may become an efficient and self-reinforcing shortcut to fulfilling entertainment and arousal needs (Schmitt, 2017), potentially contributing to problematic engagement. Complex or extreme motives may also be reflected in what has been coined “the disruptor” in linking gamification motives and extremism (Schlegel, 2021). When considering gaming's relationship to extremism, scholars have pointed to the role of humor (Askanius, 2021; Fielitz and Ahmed, 2021) and shitposting (Evans, 2019). Thus, the transmission of fun should also be considered (Lakhani, 2021) as extremist groups have leveraged internet humor cultures to normalize radical ideas under the guise of entertainment, which suggests that hedonistic gaming motives may, under specific conditions, intersect with radicalization pathways. This logic is echoed in the concept of “the disruptor” user type in linking gamification motives to extremism (Schlegel, 2021), characterized by a desire to provoke, destabilize, and gain visibility through negative attention. Disruptors may engage in trolling, doxing, or the spreading of dark-humored memes—not merely for mischief, but to achieve social recognition and status within certain in-groups. Platforms and communities that reward such behavior—sometimes even gamifying it—can deepen engagement and legitimize extremism as play. The present study considers such complex or extreme motives as part of its expanded theoretical framework to more accurately reflect potentially nuanced patterns in the data.

## 5 Linking gaming motivation to radical attitudes

Considering the ongoing debate on gaming and extremism, this study examines how motivational profiles for video game play is linked to radical attitudes. Specifically, we analyze motivational profiles as predictors of radical attitudes while incorporating known precursors of extremism as mediators. These mediators set alleged relationships in context of relevance. That is, how proximal gaming-related factors interact with factors known to increase the likelihood for radicalization. We utilize motivational profiles because, according to uses and gratification theory (Katz et al., 1973), a person may be driven by several different motives at once.

In this context, we define a set of three indicators usually associated with a radical mindset. First, conspiracy beliefs are consistently associated with both extreme-left and—more prominently—extreme-right ideological orientations (Imhoff et al., 2022, p. 392). Conspiracy mentality, a related but broader construct, is a generalized political attitude marked by distrust in power and the belief that powerful groups orchestrate harmful events (Imhoff and Bruder, 2014; Imhoff et al., 2021). Such beliefs can externalize personal frustration, fear, or uncertainty, fostering antagonistic perceptions of outgroups (Douglas et al., 2019). In group contexts, this often manifests in Manichean worldviews tied to radicalization (McCauley and Moskalenko, 2008). It can lower thresholds for radical engagement by promoting anti-system sentiments and justifying action against perceived corrupt elites (Imhoff and Bruder, 2014). In this sense, it constitutes a *conducive mindset* for radicalization—aligned with definitions of radicalism that emphasize normative or systemic change (Schmid, 2013)—without being a sufficient cause in itself. We, thus, include two prominent narratives at the time of data collection, namely a specific one concerning COVID-19 and one concerning elites in general (Schulze et al., 2022). Second, *xenophobia* represents the perceived or actual threat alien cultures have on the native in-group and is considered a core far-right belief (Carter, 2018) while implying ethnic superiority and racial gradiation (Bjørgo and Ravndal, 2019). Lastly and most importantly, we incorporate the *acceptance of political violence*, which represents a late-stage radicalization outcome (Klausen et al., 2020).

Aside conspiratorial beliefs as a potential catalyst of radicalization dynamics, research evaluated a wide array of other possible individual factors that drive people into radicalization (Schmid, 2013). Among them, most focus on the social, economic and cultural traumatic experiences an individual has faced. In this context, *marginalization* of a person is a collective factor defined as the “experience by a wide arrange of individuals and groups, examining the multiple effects of poverty and multiple deprivation upon people's and children's lives” (Mowat, 2015, p. 462). Therefore, especially young people can be affected by marginalization as it limits their life opportunities and exposes them to developing antisocial or radical attitudes (Bull and Rane, 2019). The perception of collective marginalization, where individuals feel that their in-group is systematically disadvantaged or excluded, has been empirically linked to the endorsement of anti-democratic and extremist attitudes, mediated by negative emotions (Fischer et al., 2022). Marginalization may therefore serve as an important factor influencing the effect gaming motivations might have on radical attitudes: It may represent a need violation on the collective level, and gaming can serve as a compensatory space particularly affording social inclusion.

As a complement including the individual level, *anomic insecurity* (anomie for brevity) refers to a sociopsychological, distressful state of uncertainty, disorientation, and instability that arises when individuals perceive their social environment as unpredictable or unstructured. Anomie fosters susceptibility to extremist ideologies by creating a cognitive and emotional vacuum where traditional values and social structures appear inadequate or illegitimate. The likelihood of embracing radical and extremist attitudes can be further increased if such anomic insecurity is focused on the core values, traditions, or other significant identity-forming traits of those impacted (Van den Bos, 2018).

Anomie and marginalization both increase the likelihood that individuals will seek alternative sources of meaning, and gaming, when intertwined with radical content, can become a conduit for ideological shifts. We assume that most gamers do not radicalize—as this process is not deterministic. But given past gaming-related radicalization trajectories, we aim to explore how robust a proximal relationship between gaming and extremism is through associating gaming motivations and radical attitudes in direct contrast to known precursors of radicalization.

## 6 Method

### 6.1 Participants

This study was part of a *nationwide*, quota-based online survey (CAWI) to examine democracy-distance, extremist attitudes, acceptance of politically motivated violence and intolerance toward minorities and foreign groups among young people and adolescents in Germany (Farren et al., 2023). The data collection was conducted by the field research institute Kantar (now Verian) using random sampling based on the German population registers. The survey's response rate was 31.7%. For further information, please see (Farren et al. 2023). From originally *N* = 3590 participants from which we used *N* = 2364 (=67%) individuals that indicated that they play computer games at least “rarely” and answered all motivation items. Thus, this sample reflects the attitudes and behaviors of young people who engage with digital games regularly, which may influence the generalizability of the findings to the broader population of young people in Germany. The sample consisted of 59% men, 39% women and 2% divers. They were between 16 and 22 years old (*M* = 18.58, MD = 19, SD = 1.76). Most participants have or are aiming for a general higher education entrance qualification/vocational baccalaureate (74%), 22% had an intermediate school leaving certificate, 3% had secondary school certificate, and 1% had no general school leaving certificate. Participants reported their frequency how often they used computer, video games or digital games (e.g., mobile, computer, and console games) for entertainment in the past 4 weeks on a scale from 1 (never) to 5 (very frequently). The distribution showed that 13.2% of participants reported playing rarely, 30.4% reported playing sometimes, 24.5% reported playing frequently, and 31.9% reported playing very frequently.

### 6.2 Measurement

Responses for marginalization, anomie, and radical attitudes were recorded on a four-point scale (1 = strongly disagree to 4 = strongly agree). Full details on these measures can be found in (Farren et al. 2023). We assessed collective experiences of marginalization with three items, asking participants to indicate their agreement with the statements: (1) “Here with us, people like me are often held in low esteem by others,” (2) “Here with us, people like me are not taken seriously by politicians,” and (3) “Here with us, people like me are treated unfairly by the police.” Anomie was measured with three items assessing perceived societal instability and uncertainty: (1) “These days, everything has become so uncertain that one must be prepared for anything,” (2) “When looking at the events of recent years, one becomes truly insecure,” and (3) “Things have become so difficult today that one no longer knows what is going on.”

Radical attitudes included xenophobia and two types of stances toward conspiracy narratives, namely anti-elite narratives and COVID-19-related narratives. Xenophobia was measured with two items: (1) “There are too many foreigners in Germany,” and (2) “Most foreigners living in Germany do not follow the common rules here.” Belief in anti-elite conspiracy theories was assessed with two items: (1) “There are secret organizations that have great influence on political decisions,” and (2) “Politicians and other leaders are only puppets of the powers behind them.” Similarly, belief in COVID-19-related conspiracies was captured using two items: (1) “The true origin of the Coronavirus is deliberately kept secret by our government,” and (2) “The dangerous side effects of vaccinations are deliberately concealed.” Acceptance of political violence was assessed with four items: (1) “In every democratic society, there are certain conflicts that must be resolved through violence,” (2) “The necessary changes in this society can only be brought about through a violent revolution,” (3) “Sometimes, one must defend oneself against representatives of the system with violence,” and (4) “Even in a democracy, it is sometimes necessary to use violence to achieve political goals.”

Gaming motivation was assessed using ten binary (yes/no) items, adapted from The Motives for Online Gaming Questionnaire (MOGQ; Demetrovics et al., 2011). All items were introduced with “I use these games…”. Entertainment and recreation motives were captured with two items: (1) “…because it is fun,” and (2) ”…to pass the time.” Social motives were assessed with two items: (1) “… to be in company or with friends,” and (2) “...to meet new or interesting people.” Competition motives were measured with two items: (1) “...because I like to win,” and (2) “...because I enjoy defeating others.” Escapism/coping motives were assessed using three items: (1) “...because they make me forget about real life,” (2) “...to get away from my everyday problems,” and (3) “...to get rid of stress, fear, or negative emotions.” Additionally, we included one item assessing motivations to increase social status through gaming: “...to be appreciated by my friends and to be considered an expert.”

Participants indicated in an open question what game they mainly played (Which computer or video games are you currently playing? The game I play most frequently: …” and “Additionally, I also play: …”). Top five primarily played games were FIFA series (~7% share), League of Legends (LoL; ~5%), Valorant (~4%), Hay Day (~4%), Minecraft (~4%). This was used to build participants' genre preferences adapting a schema used in (Kowert et al. 2022), including *simulation and sports*, the most frequently played genre (28.31%), followed by *multiplayer online battle arena (MOBA)* games (18.46%), *puzzle and party games* (8.75%), *shooter* (7.75%), *action-adventure* (8.27%), *sandbox* (4.93%), *real-time strategy* (6.55%), and *action-roleplaying* (5.69%). Less commonly played genres were *multiplayer shooter* (2.44%), *survival and horror* (2.10%), *Action-roleplay* (1.67%), and *platformer* (2.30%). Additionally, 2.77% of responses were categorized as *other*.

### 6.3 Statistical analysis

Our approach to the analysis of the current data was two-fold: First, we performed latent class analysis (LCA) to inductively examine latent motivational types of gaming motivation by modeling latent classes. LCA assumes that heterogeneous variables can be represented by homogeneous and mutually exclusive categorical latent variables or clusters (Collins and Lanza, 2009). LCA is suited for binary response variables and attributes cases to the value of the categorical, underlying latent variable it most probably belongs to (Karnowski 2017). The decision for the best clustering solution can be informed by the Bayesian information criterion (BIC) (Karnowski, 2017). In general, the solution with the lowest BIC can be considered the most suitable.

Second, we used these clusters within a series of mediation analyses, one for each profile as a predictor. This allowed us to decompose the total effects (the effects that include predictor as well as mediator) of our profiles on radicalization outcomes into a direct (predictor's sole effect on the outcome variable) and an indirect (predictor's effect mediated via marginalization and anomie) effect. The latter is further broken down into *path a* (effect of predictor on mediators) and *path b* (effect of mediators on outcome, see Table 1). We examined the potential direct effects of each of these motivational profiles on several radical outcomes (such as xenophobia) or whether this effect is generated by known precursors of extremism, such as marginalization. All mediations are controlled for education, sex, video-game usage, and a political left-right self-positioning. Mediation analysis was performed within structural equation models to include target variables as latent constructs.

**Table 1:** Overview of all path coefficients and effects of the mediation analysis.

**Profile**	**Effect**	**Variable**	**Estimate**	**CI lower**	**CI upper**
**The Escapist**	Path a	Anomie	0.09^***^	0.05	0.14
	Path a	Marginalization	0.01	−0.04	0.06
	Direct effect	Acceptance of political violence	−0.02	−0.06	0.03
	Direct effect	Anti-elite conspiracy belief	−0.04	−0.08	0.00
	Direct effect	COVID-19 conspiracy belief	−0.07^***^	−0.12	−0.03
	Direct effect	Xenophobia	−0.06^**^	−0.11	−0.02
	Total indirect effect	Acceptance of political violence	0.00	−0.01	0.02
	Total indirect effect	Anti-elite conspiracy belief	0.02	−0.00	0.04
	Total indirect effect	COVID-19 conspiracy belief	0.02	−0.00	0.04
	Total indirect effect	Xenophobia	0.02^*^	0.00	0.03
	Total effect	Acceptance of political violence	−0.02	−0.06	0.03
	Total effect	Anti-elite conspiracy belief	−0.02	−0.06	0.03
	Total effect	COVID-19 conspiracy belief	−0.05^*^	−0.10	−0.01
	Total effect	Xenophobia	−0.05^*^	−0.09	−0.00
**The competitive-escapist**	Path a	Anomie	0.02	−0.03	0.07
	Path a	Marginalization	0.08^**^	0.03	0.13
	Direct effect	Acceptance of political violence	0.03	−0.02	0.07
	Direct effect	Anti-elite conspiracy belief	0.05^*^	0.01	0.10
	Direct effect	COVID-19 conspiracy belief	0.04	−0.01	0.08
	Direct effect	Xenophobia	−0.01	−0.05	0.04
	Total indirect effect	Acceptance of political violence	0.03^**^	0.01	0.04
	Total indirect effect	Anti-elite conspiracy belief	0.03^**^	0.01	0.05
	Total indirect effect	COVID-19 conspiracy belief	0.03^**^	0.01	0.05
	Total indirect effect	Xenophobia	0.01^*^	0.00	0.03
	Total effect	Acceptance of political violence	0.05^*^	0.01	0.10
	Total effect	Anti-elite conspiracy belief	0.08^***^	0.04	0.13
	Total effect	COVID-19 conspiracy belief	0.07^**^	0.02	0.12
	Total effect	Xenophobia	0.01	−0.04	0.05
**The social-escapist**	Path a	Anomie	0.10^***^	0.05	0.15
	Path a	Marginalization	0.08^**^	0.02	0.13
	Direct effect	Acceptance of political violence	0.03	−0.02	0.08
	Direct effect	Anti-elite conspiracy belief	−0.04^*^	−0.09	−0.00
	Direct effect	COVID-19 conspiracy belief	−0.02	−0.06	0.03
	Direct effect	Xenophobia	−0.00	−0.04	0.04
	Total indirect effect	Acceptance of political violence	0.03^**^	0.01	0.04
	Total indirect effect	Anti-elite conspiracy belief	0.05^***^	0.02	0.07
	Total indirect effect	COVID-19 conspiracy belief	0.04^***^	0.02	0.07
	Total indirect effect	Xenophobia	0.03^***^	0.01	0.04
	Total effect	Acceptance of political violence	0.05^*^	0.00	0.10
	Total effect	Anti-elite conspiracy belief	0.00	−0.04	0.05
	Total effect	COVID-19 conspiracy belief	0.03	−0.02	0.07
	Total effect	Xenophobia	0.03	−0.02	0.07
**The competitor**	Path a	Anomie	−0.10^***^	−0.16	−0.05
	Path a	Marginalization	−0.07^**^	−0.12	−0.03
	Direct effect	Acceptance of political violence	0.01	−0.03	0.06
	Direct effect	Anti-elite conspiracy belief	0.06^**^	0.01	0.10
	Direct effect	COVID-19 conspiracy belief	0.03	−0.01	0.07
	Direct effect	Xenophobia	0.00	−0.04	0.05
	Total indirect effect	Acceptance of political violence	−0.02^**^	−0.04	−0.01
	Total indirect effect	Anti-elite conspiracy belief	−0.05^***^	−0.07	−0.02
	Total indirect effect	COVID-19 conspiracy belief	−0.04^***^	−0.07	−0.02
	Total indirect effect	Xenophobia	−0.03^***^	−0.04	−0.01
	Total effect	Acceptance of political violence	−0.01	−0.06	0.03
	Total effect	Anti-elite conspiracy belief	0.01	−0.04	0.06
	Total effect	COVID-19 conspiracy belief	−0.01	−0.06	0.03
	Total effect	Xenophobia	−0.03	−0.07	0.02
**The absorber**	Path a	Anomie	0.08^**^	0.03	0.13
	Path a	Marginalization	0.10^***^	0.06	0.15
	Direct effect	Acceptance of political violence	0.06^*^	0.01	0.11
	Direct effect	Anti-elite conspiracy belief	0.03	−0.01	0.08
	Direct effect	COVID-19 conspiracy belief	0.00	−0.04	0.05
	Direct effect	Xenophobia	0.03	−0.02	0.08
	Total indirect effect	Acceptance of political violence	0.03^***^	0.02	0.05
	Total indirect effect	Anti-elite conspiracy belief	0.05^***^	0.03	0.08
	Total indirect effect	COVID-19 conspiracy belief	0.05^***^	0.03	0.07
	Total indirect effect	Xenophobia	0.03^***^	0.01	0.04
	Total effect	Acceptance of political violence	0.10^***^	0.04	0.15
	Total effect	Anti-elite conspiracy belief	0.09^***^	0.04	0.13
	Total effect	COVID-19 conspiracy belief	0.05^*^	0.01	0.10
	Total effect	Xenophobia	0.06^*^	0.01	0.10
**The socializer**	Path a	Anomie	−0.06^*^	−0.11	−0.01
	Path a	Marginalization	−0.09^***^	−0.14	−0.04
	Direct effect	Acceptance of political violence	−0.06^**^	−0.09	−0.02
	Direct effect	Anti-elite conspiracy belief	−0.01	−0.05	0.04
	Direct effect	COVID-19 conspiracy belief	−0.02	−0.06	0.03
	Direct effect	Xenophobia	−0.00	−0.04	0.04
	Total indirect effect	Acceptance of political violence	−0.03^***^	−0.05	−0.01
	Total indirect effect	Anti-elite conspiracy belief	−0.05^***^	−0.07	−0.02
	Total indirect effect	COVID-19 conspiracy belief	−0.04^***^	−0.07	−0.02
	Total indirect effect	Xenophobia	−0.02^***^	−0.04	−0.01
	Total effect	Acceptance of political violence	−0.09^***^	−0.13	−0.05
	Total effect	Anti-elite conspiracy belief	−0.05^*^	−0.10	−0.01
	Total effect	COVID-19 conspiracy belief	−0.06^**^	−0.10	−0.02
	Total effect	Xenophobia	−0.02	−0.07	0.02
**The recreationalist**	Path a	Anomie	−0.10^***^	−0.15	−0.05
	Path a	Marginalization	−0.09^***^	−0.14	−0.04
	Direct effect	Acceptance of political violence	−0.06^*^	−0.10	−0.01
	Direct effect	Anti-elite conspiracy belief	−0.05^*^	−0.09	−0.01
	Direct effect	COVID-19 conspiracy belief	0.02	−0.02	0.07
	Direct effect	Xenophobia	0.03	−0.01	0.08
	Total indirect effect	Acceptance of political violence	−0.03^**^	−0.05	−0.01
	Total indirect effect	Anti-elite conspiracy belief	−0.05^***^	−0.08	−0.03
	Total indirect effect	COVID-19 conspiracy belief	−0.05^***^	−0.07	−0.03
	Total indirect effect	Xenophobia	−0.03^***^	−0.04	−0.02
	Total effect	Acceptance of political violence	−0.09^***^	−0.13	−0.04
	Total effect	Anti-elite conspiracy belief	−0.10^***^	−0.15	−0.05
	Total effect	COVID-19 conspiracy belief	−0.03	−0.07	0.02
	Total effect	Xenophobia	0.00	−0.04	0.05

Estimate, standardized coefficients; CI, Confidence Interval; ^*^p < 0.05; ^**^p < 0.01; ^***^p < 0.001.

All analyses were carried out within the R statistical environment (R Core Team, 2022). For LCA we used the package “polLCA” (Linzer and Lewis, 2011) and for structural equation modeling we used the package “lavaan” (Rosseel, 2012). For both the LCA and SEM analyses, we relied on listwise deletion of cases with missing values on relevant variables. While the sample size remained unaffected in the LCA, SEM models are based on 2247 complete cases out of 2342 (approx. additional 4.1% excluded).

## 7 Results

### 7.1 Gaming motivation profiles—latent class analysis

**Figure 2:**
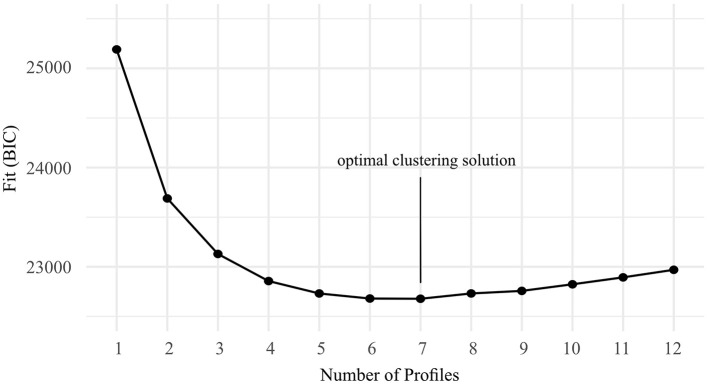
Evaluation of clustering solutions based on Bayesian Information Criterion (BIC). The lowest point indicates that a 7-profile solution provides the optimal trade-off between model complexity and fit.

In our case, a seven-cluster solution showed the best fit (*BIC* = 22677.62, see Figure 2) as it presents the lowest BIC value across the scale of possible cluster solutions.

**Figure 3:**
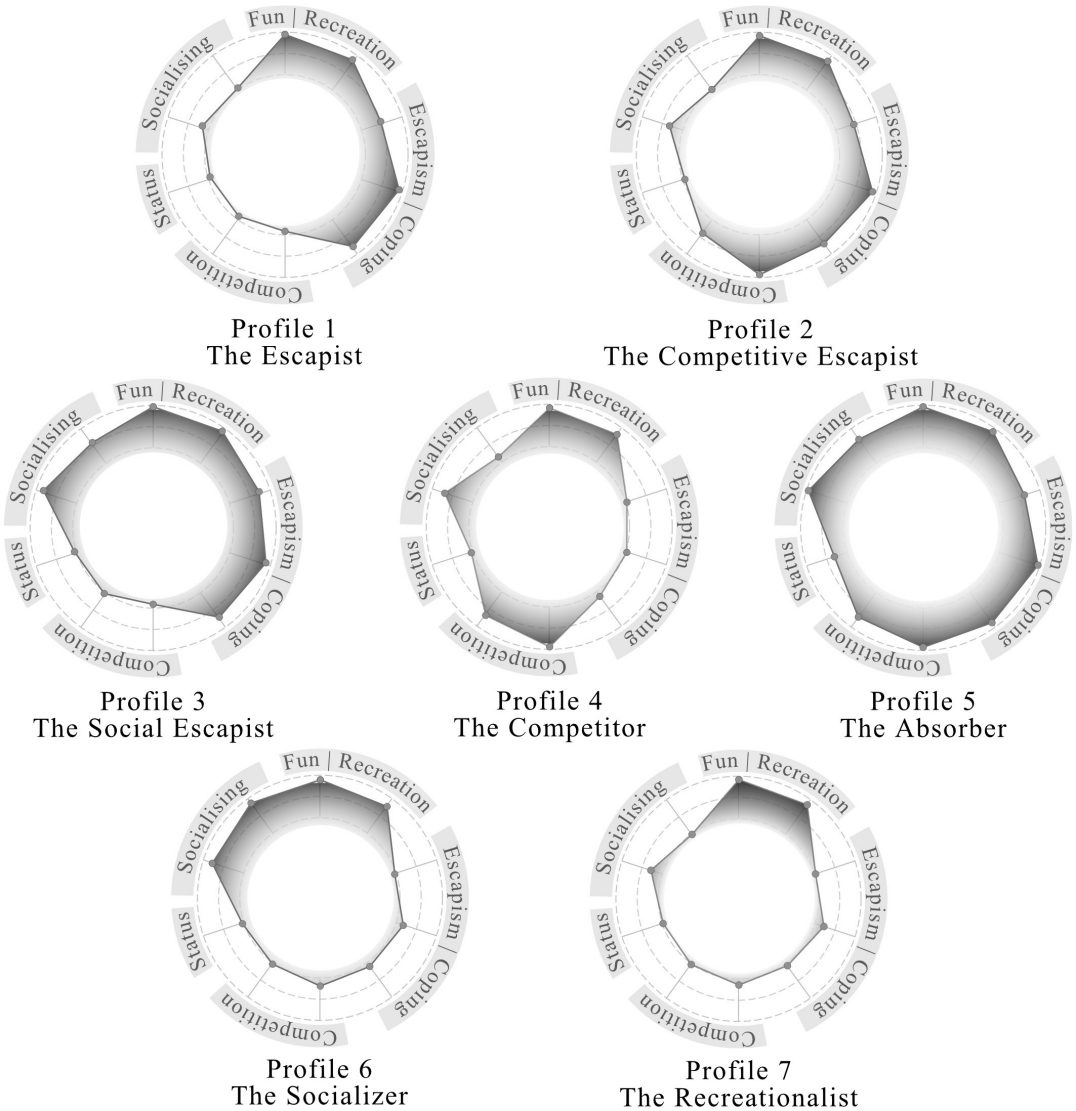
Radar plots of gaming motivations across the seven latent gamer profiles derived from Latent Class Analysis. Each axis represents response rates to a binary motivation item (e.g., “because it is fun”, “to compete”, etc.), and the shaded area shows the proportion of participants within each profile who endorsed the respective motive (range: 0–1). Labels group items by thematic clusters (e.g., Fun/Recreation, Escapism, Competition) to aid interpretability.

The resulting profiles showed an overall balanced distribution across our sample. It is notable that hedonic motives were present in all of the seven motivation profiles. Specific profile characteristics are given below (see Figure 3 and Supplementary material).

#### 7.1.1 Profile 1: the escapist

In this gaming motivation profile, the escapism/coping motive was dominant, showing almost no associations with competitive, and more crucially, social motives. The overall sample share of profile 1 is 13.2%, with a gender share of 37% men, 60% women, and 3% diverse. Participants in profile 1 show a preference for simulation and sports games and consider themselves casual to frequent gamers (*M* = 3.54, *MD* = 3, *SD* = 0.97).

#### 7.1.2 Profile 2: the competitive-escapist

In this gaming motivation profile, the escapism/coping motive was prominent but accompanied by moderate motives to compete as well as comparably weak social motives. The overall sample share of profile 2 is 13.2%, with a gender share of 61% men, 38% women, and < 1% diverse. Participants in profile 2 show mixed preferences for the genre simulation and sports (27%) and considered themselves as mostly frequent gamers (*M* = 3.87, *MD* = 4, *SD* = 0.97).

#### 7.1.3 Profile 3: the social-escapist

In this profile, the escapism/coping motive was again prominent but accompanied by firm social motives in the absence of motives to compete. The overall sample share of profile 3 is 12.4%, with a gender share of 57% men, 40% women, and 3% diverse. Participants in profile 3 showed mixed preferences with a tendency for simulation and sports (23%) games and considered themselves frequent gamers (*M* = 4.04, *MD* = 4, *SD* = 0.95).

#### 7.1.4 Profile 4: the competitor

In this gaming motivation profile, the competition motive was dominant, accompanied by selective social motives while escapism motives were absent. The overall sample share of profile 4 is 14%, with a gender share of 80% men, 18% women, and 2% diverse. The most frequently reported gaming genre in profile 4 was multiplayer online battle arena (21%). Profile 4 players considered themselves mostly frequent gamers (*M* = 3.83, *MD* = 4, *SD* = 1.03).

#### 7.1.5 Profile 5: the absorber

In this gaming motivation profile, all motives are present. It is also the profile with the highest probability to use video-gaming for increasing social-status. The overall sample share of profile 5 is 12.3%, with a gender share of 73% men, 24% women, and 3% diverse. The most frequent reported gaming genre in profile 5 was multiplayer online battle arena (31%). Players considered themselves frequent to very frequent gamers (*M* = 4.30, *MD* = 5, *SD* = 0.91).

#### 7.1.6 Profile 6: the socializer

In this profile, the social motive is exclusively dominant. Other motives are virtually absent, including the social-status motive. The overall sample share of profile 6 is 9.1%, with a gender share of 68% men, 31% women, and 1% diverse. Participants in profile 6 showed a preference for genre simulation and sports (24%) and considered themselves frequent gamers (*M* = 4.06, *MD* = 4, *SD* = 1.02).

#### 7.1.7 Profile 7: the recreationalist

In this gaming motivation profile, fun and distraction motives dominate in the virtual absence of other motives. Profile 7 had the largest sample share with 25.8%, with a gender share of 50% men, 50% women, and < 1% diverse. Participants in profile 7 showed a preference for genre simulation and sports (30%) and considered themselves mostly casual gamers (*M* = 3.23, *MD* = 3, *SD* = 0.99).

### 7.2 Mediation analysis

**Figure 4:**
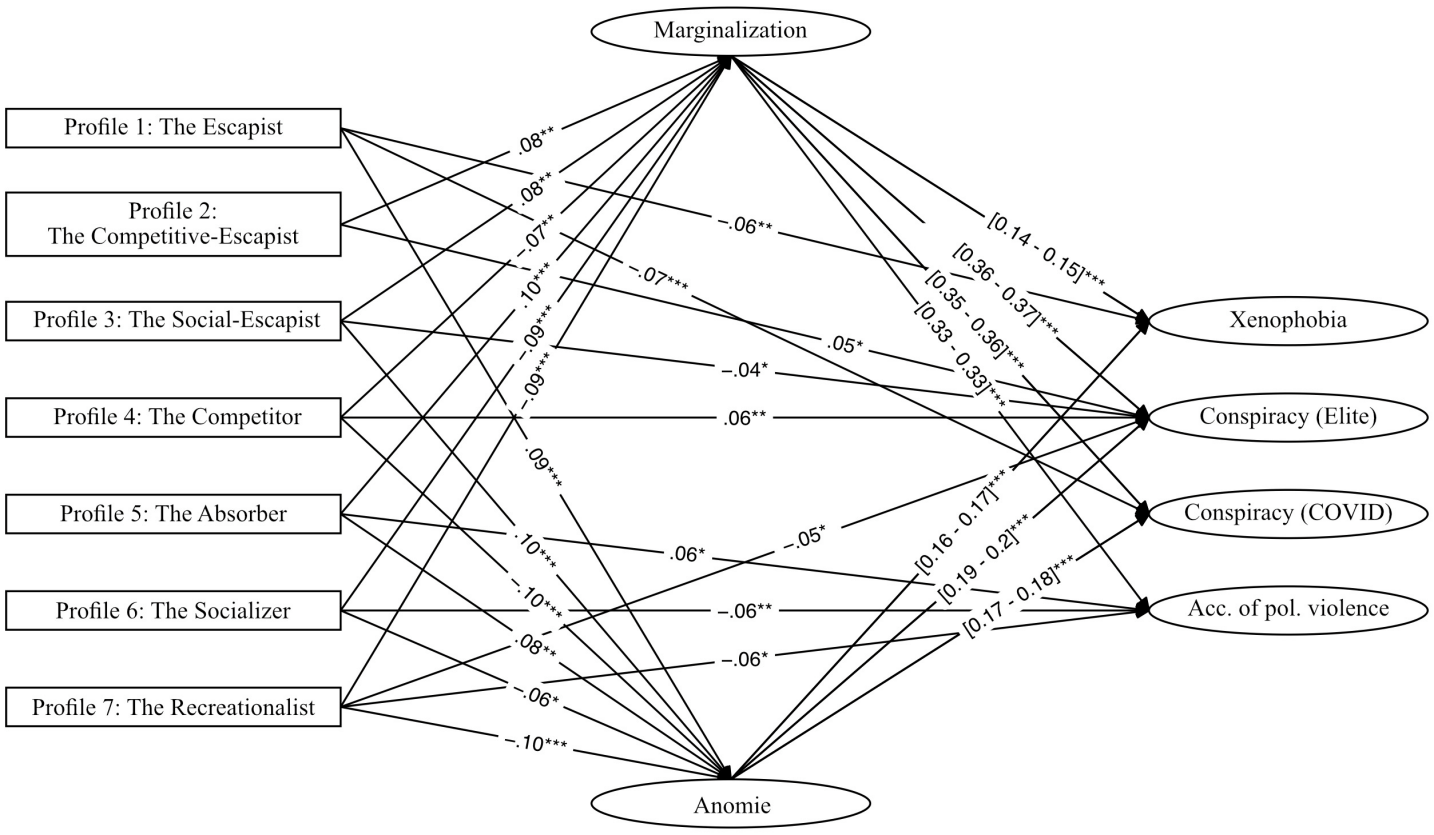
Structural model displaying significant standardized direct effects from latent gamer profiles to radical outcome variables, mediated by marginalization and anomie. **Arrows** represent only statistically significant paths (*p* < 0.05) to reduce visual complexity. **Numerical values** indicate standardized regression coefficients; for paths from mediators to outcomes, coefficient ranges are shown in **brackets**. Significance levels are marked as follows: *p < 0.05; **p < 0.01; ***p < 0.001.

The established structural equation models demonstrated a good model fit across all profiles (χ^2^_(147)_ = [207.24–214.56], *ps* < 0.01, CFIs ≥ 0.99, RMSEAs ≤ 0.02, SRMRs ≤ 0.02, TLIs ≥ 0.99). The direct effects of the mediators marginalization and anomie on radical outcomes were substantial and robustly positive across all radical indices and models. Marginalization significantly predicted xenophobia (β = [0.14–0.15], ps < 0.001), anti-elite conspiracy belief (β = [0.36–0.37], *ps* < 0.001), COVID-19 conspiracy belief (β = [0.35–0.36], *ps* < 0.001), and political violence (β = 0.33, *ps* < 0.001), validating marginalization as a powerful precursor of radical attitudes. Similarly, anomie was a significant predictor of xenophobia (β = [0.16–0.17], *ps* < 0.001), anti-elite conspiracy belief (β = [0.19–0.2], *ps* < 0.001), and COVID-19 conspiracy belief (β = [0.17–0.18], *p* < 0.001), while it did not predict political violence (*p* > 0.05). For an overview, see Figure 4 and Table 1.

#### 7.2.1 Profile 1: the escapist

The Escapist was characterized by a significant positive effect on anomie (β = 0.09, *p* < 0.001), indicating that individuals with escapist gaming motives tend to experience heightened anomie—a sense of social disconnection or normlessness. In contrast, marginalization was not significantly predicted by this profile (β = 0.01, *p* > 0.05), suggesting that escapists do not perceive themselves as socially excluded.

In terms of direct effects, escapists showed a significant negative association with xenophobia (β = −0.06, *p* < 0.01) and COVID-19 conspiracy beliefs (β = −0.07, *p* < 0.001), implying a reduced tendency toward these radical attitudes. No significant direct effects emerged for acceptance of political violence or anti-elite conspiracy beliefs (*p* > 0.05).

Although total indirect effects were small, there was a weak but significant indirect effect on xenophobia (β = 0.02, *p* < 0.05), suggesting that the combined influence of anomie and marginalization slightly contributed to increased xenophobic attitudes. However, no other total indirect effects were observed, indicating that neither anomie nor marginalization strongly mediate radicalization processes for this group.

Overall, players with escapist gaming motives exhibit a mixed pattern: while they are more prone to experiencing anomie, they tend to reject xenophobia and conspiracy beliefs, suggesting that escapism itself is not inherently tied to radicalization. Instead, these players may use gaming as a means of psychological detachment rather than an avenue for reinforcing extremist attitudes.

#### 7.2.2 Profile 2: the competitive-escapist

Competitive Escapists were significantly more likely to experience marginalization (β = 0.08, *p* < 0.01), indicating that players with a strong competitive-escapist motivation tend to perceive themselves as socially excluded. In contrast to escapists, this profile was not significantly associated with anomie (β = 0.02, *p* > 0.05). Regarding direct effects, Competitive Escapists showed a small but significant positive association with anti-elite conspiracy beliefs (β = 0.05, *p* < 0.05). No significant direct effects were observed for xenophobia, COVID-19 conspiracy beliefs, or acceptance of political violence (*p* > 0.05).

Total indirect effects revealed significant mediation through marginalization and anomie for all radical outcomes, including xenophobia (β = 0.01, *p* < 0.05), anti-elite conspiracy beliefs (β = 0.03, *p* < 0.01), COVID-19 conspiracy beliefs (β = 0.03, *p* < 0.01), and acceptance of political violence (β = 0.03, *p* < 0.01). These findings suggest that while Competitive Escapists do not strongly endorse radical beliefs on their own, their experience of marginalization contributes to the adoption of these attitudes.

The total effects underscore this pattern: Competitive Escapists showed significant positive associations with acceptance of political violence (β = 0.05, *p* < 0.05), anti-elite conspiracy beliefs (β = 0.08, *p* < 0.001), and COVID-19 conspiracy beliefs (β = 0.07, *p* < 0.01). These results point to partial mediation, as indirect effects via marginalization account for much of the associations to radical outcomes.

Overall, Competitive Escapists demonstrate a meaningful, but weak inclination toward radical attitudes. While direct effects are limited, the presence of multiple indirect pathways highlights the role of marginalization in facilitating these beliefs. This suggests that competitive gaming motivations may not increase the likelihood of adopting radical attitudes, but social exclusion may act as an amplifying factor.

#### 7.2.3 Profile 3: the social-escapist

Social Escapists were significantly more likely to experience both marginalization (β = 0.08, *p* < 0.01) and anomie (β = 0.10, *p* < 0.001), suggesting that this player profile is associated with feelings of social exclusion and normlessness.

Regarding direct effects, Social Escapists exhibited a small but significant negative association with anti-elite conspiracy beliefs (β = −0.04, *p* < 0.05), indicating a reduced likelihood of endorsing such attitudes. However, no significant direct effects emerged for xenophobia, COVID-19 conspiracy beliefs, or acceptance of political violence.

Total indirect effects were significant for all radical attitudes, including xenophobia (β = 0.03, *p* < 0.001), anti-elite conspiracy beliefs (β = 0.05, *p* < 0.001), COVID-19 conspiracy beliefs (β = 0.04, *p* < 0.001), and acceptance of political violence (β = 0.03, *p* < 0.01). These results indicate that marginalization and anomie mediate the relationship between Social Escapism and radical attitudes.

Total effects showed only small but significant association between Social Escapists and acceptance of political violence (β = 0.05, *p* < 0.05).

Overall, Social Escapists do not exhibit direct inclinations toward radical attitudes. Instead, their experiences of marginalization and anomie contribute to radical beliefs in an indirect manner, pointing to a largely to fully mediated relationship. This suggests that escapism with social motivations does not inherently lead to radicalization, but social exclusion and feelings of normlessness may facilitate these attitudes.

#### 7.2.4 Profile 4: the competitor

Competition-oriented players were significantly less likely to experience marginalization (β = −0.07, *p* < 0.01) and anomie (β = −0.10, *p* < 0.001). They showed no significant direct effects on radical attitudes, except for a small positive association with anti-elite conspiracy beliefs (β = 0.06, *p* < 0.01). However, indirect effects were negatively significant across all radical outcomes (xenophobia: β = −0.03, *p* < 0.001; anti-elite conspiracy belief: β = −0.05, *p* < 0.001; COVID-19 conspiracy belief: β = −0.04, *p* < 0.001; acceptance of political violence: β = −0.02, *p* < 0.01).

Although marginalization is typically a strong predictor of radical attitudes, competition-oriented players were less likely to experience it, thereby leading to a suppressor effect—strengthening the negative relationship between competition motivation and radical attitudes. This suggests that competitively motivated players may be indirectly shielded from radicalization due to lower exposure to marginalization and anomie.

#### 7.2.5 Profile 5: the absorber

Players classified as absorbers exhibited the highest likelihood of experiencing marginalization (β = 0.10, *p* < 0.001) and anomie (β = 0.08, *p* < 0.01) compared to all other groups. They also showed a direct positive relationship with acceptance of political violence (β = 0.06, *p* < 0.05), while direct effects on other radical attitudes remained non-significant.

All indirect effects were positively significant (xenophobia: β = 0.03, *p* < 0.001; anti-elite conspiracy belief: β = 0.05, *p* < 0.001; COVID-19 conspiracy belief: β = 0.05, *p* < 0.001; acceptance of political violence: β = 0.03, *p* < 0.001), suggesting that marginalization and anomie function as mediators for radical attitudes.

Total effects confirmed substantial positive associations with all radical outcomes (xenophobia: β = 0.06, *p* < 0.05; anti-elite conspiracy belief: β = 0.09, *p* < 0.001; COVID-19 conspiracy belief: β = 0.05, *p* < 0.05; acceptance of political violence: β = 0.10, *p* < 0.001). The combination of a direct link to political violence and strong indirect as well as total effects suggests that this profile has the most pronounced connection to radical attitudes, with a large portion of this effect being mediated through marginalization.

#### 7.2.6 Profile 6: the socializer

Individuals with dominant social gaming motives exhibited a significantly lower likelihood of experiencing marginalization (β = −0.09, *p* < 0.001) and anomie (β = −0.06, *p* < 0.05). Furthermore, they showed a negative direct effect on acceptance of political violence (β = −0.06, *p* < 0.01), while direct effects on other radical attitudes were non-significant.

Total indirect effects were significantly negative across all radical measures (xenophobia: β = −0.02, *p* < 0.001; anti-elite conspiracy belief: β = −0.05, *p* < 0.001; COVID-19 conspiracy belief: β = −0.04, *p* < 0.001; acceptance of political violence: β = −0.03, *p* < 0.001), indicating that the association between social gaming motives and radical attitudes is mediated through marginalization and anomie in a suppressing manner.

Total effects confirmed an overall negative relationship with all radical outcomes, particularly for acceptance of political violence (β = −0.09, *p* < 0.001), anti-elite conspiracy belief (β = −0.05, *p* < 0.05), and COVID-19 conspiracy belief (β = −0.06, *p* < 0.01). These findings suggest that players with a strong social gaming motivation tend to be negatively associated with radical attitudes, with the mediation of marginalization reinforcing this pattern.

#### 7.2.7 Profile 7: the recreationalist

Players with a dominant hedonistic gaming motivation were significantly less likely to experience anomie (β = −0.10, *p* < 0.001) and marginalization (β = −0.09, *p* < 0.001). Additionally, they exhibited direct negative effects on acceptance of political violence (β = −0.06, *p* < 0.05) and anti-elite conspiracy belief (β = −0.05, *p* < 0.05), while direct effects on COVID-19 conspiracy belief and xenophobia remained non-significant.

Total indirect effects were significantly negative across all radical measures (xenophobia: β = −0.03, *p* < 0.001; anti-elite conspiracy belief: β = −0.05, *p* < 0.001; COVID-19 conspiracy belief: β = −0.05, *p* < 0.001; acceptance of political violence: β = −0.03, *p* < 0.01), suggesting that marginalization and anomie function as suppressors in the relationship between hedonistic gaming motivation and radical attitudes.

Total effects further confirmed a broad negative association between this profile and radical attitudes, particularly for anti-elite conspiracy belief (β = −0.10, *p* < 0.001) and acceptance of political violence (β = −0.09, *p* < 0.001). No significant total effect emerged for COVID-19 conspiracy belief or xenophobia, suggesting that the negative influence of hedonistic gaming motivation on radical attitudes is mostly indirectly mediated through marginalization and anomie rather than exerting strong direct effects.

## 8 Discussion

The question of how gaming and the gaming ecosystem contribute to radicalization processes is still discussed in public, scientific, and political discourse. From a distal-proximal perspective, the influence of gaming on radicalization can stem from both external community dynamics and direct individual engagement with games. While distal factors, such as extremist networking within gaming communities, have received increasing attention, proximal factors—those tied to personal gaming experiences and motivations—remain underexplored. We address this gap by examining gaming motivation profiles as proxies for the underlying needs of players and their relationship with radical outcomes, studying under what conditions this connection may thrive. Since gaming can fulfill basic human needs (Ryan et al., 2006), and the frustration of these needs—rather than frustration with the game itself—can increase susceptibility to radicalization processes (Kruglanski et al., 2019), gaming motivations may serve as a good indicator for when gaming might lead individuals down the wrong path—just as certain motivations are more prone to indicate addictive gaming tendencies. Because the sole impact of gaming on radicalization seems questionable, we include mediating variables—experiences of collective marginalization and anomie—to contrast gaming motivations with strong precursors of radicalization pathways. Our results are suggestive against a generalized effect of gaming motivations on radical outcomes, but we nevertheless find weak associations between them under certain circumstances. The following section splits the discussion into contributing and non-contributing motivational constitutions of individuals.

The strongest inclination toward radical outcomes seems to be in participants in profile 5 (the Absorber), who indicated most of the surveyed motivations simultaneously, thus showing multi-layered drivers behind playing video games. They have the highest probability of having experienced marginalization and of advocating political violence, finally exhibiting a firm proneness toward all measured radical outcomes when facilitated through marginalization. In a need-based view of radicalization, this does not seem entirely surprising. We termed this motivational profile (5) *the absorber* because it connects diverse needs with heavy gaming use. The presence of all motives, particularly the motive to use video gaming to increase social status—virtually absent in all other motivational profiles—may reflect a more expansive role of gaming in the lives of these individuals. Previous literature has suggested that when gaming serves as a primary avenue for fulfilling diverse psychological needs, it may be associated with unmet expectations and frustration, potentially creating reinforcing cycles of high engagement and dissatisfaction (T'ng et al., 2022). While our data do not assess mental health or problematic gaming directly, high-frequency play and motivational breadth, as observed in this profile, have been linked in past research to maladaptive outcomes such as overreliance on gaming for self-esteem or identity regulation (King and Delfabbro, 2016). These considerations remain speculative in our context but may inform future investigations into the relationship between complex gaming motives and vulnerability to radical attitudes.

Proceeding to mediating factors, several profiles entail participants with strong to partial escapist basic motivations. Individuals in Profile 2 (the Competitive-Escapist) play video games primarily for escapism/coping, as well as for competitive motives. This group is associated with marginalization and anti-elite conspiracy belief, exhibiting stronger associations with radical outcomes through the mediating effect of marginalization (but not anomie). Profile 3 (Social-Escapists), in which escapism/coping motives team up with social game-play motives, suggests no direct association with radical outcomes in the first place. Both types complement the escapism/coping motive with just one other dominant motive, while escapism/coping is the common denominator of all three noticeable profiles. In combination with escapism, social motives seem to slightly dampen the effect of marginalization and the association with anti-elite conspiracy beliefs present in Profile 2. The result is that Profile 3 shows no direct effects on radical attitudes, in fact, if anything, negative ones (such as anti-elite conspiracy beliefs).

Considering the partially escapist Profiles (Competitive Escapists) 2, 3 (Social Escapists), and profile 5 (Absorbers) are frequently discussed for its associations with negative psychosocial outcomes (Tang et al., 2020; Wang et al., 2021), it is surprising that Profile 1 (The Escapist), in which mainly escapism/coping motives prevailed, didn't incline toward any of the measured radical attitudes. In fact, escapists tend to show lower levels of xenophobia and are less likely to engage with COVID-19 conspiracy narratives. It was also the only profile that remained completely unaffected by marginalization experiences, invalidating the view that suggested external dispositions for radicalization such as marginalization have a generalized impact. This is inconsistent with what we might expect from previous literature, in which negative effects of escapism are substantial. Yet, in line with expectations, anomie was linked to the escapist Profile 1. Escapism and coping motives are associated with diverse negative psychosocial outcomes, such as depression and anxiety (Wang et al., 2021), as well as narcissism, Machiavellianism, and psychopathy (Tang et al., 2020). In the current study, escapism's allegedly direct negative effects seem to be present only when in conglomerate with other motives, such as competition.

About competition-related motives, the findings of dominant competition motives as in Profile 4 (the Competitor), which were negatively inclined toward marginalization as well as anomie, and directly positively linked only to anti-elite conspiracy beliefs, seem partially at odds with prior work. The results suggest that independent of marginalization or anomie, competitive players may be slightly inclined toward anti-elite conspiratorial thinking—possibly reflecting a heightened sensitivity to hierarchical structures, perceived injustices, or opposition to external authorities.

Competitive gaming motives further seem associated with a reduced sense of social disconnection and uncertainty—factors that often drive radicalization, turning indirect effects via marginalization and anomie to be negative. Our data suggest that competition may even be a protective motive against marginalization and anomie. Rather than perceiving themselves as victims of societal exclusion, they may instead focus on personal achievement and competition, reducing the likelihood of adopting radical beliefs via marginalization/anomie. However, the direct effect on anti-elite conspiracy beliefs suggests that competitiveness may still come with an oppositional stance toward authority, warranting further investigation into the nuanced relationship between competition, social comparison, and distrust in elites. Yet, in the light of continuous reports of concerning spheres of toxic masculinity and the mix of a high proportion of men and strong competition in Profile 4, an anticipated broader inclination toward radical attitudes is not backed by our data. Future studies should examine the psychological profile of competition-oriented gamers, and whether this preference comes from a self-selection dynamic (e.g., “I prefer competitive games because I am good at it”).

Gaming out of social (Profile 6, the Socializer) and/or recreational motivations (Profile 7, the Recreationalist) seems evenly unlikely to show any radical resentments. As recreationalists seemed to form a group of fairly “for fun” gamers, other expectations would be misguided. Consistent with the existing literature, our observation indicates that social game-play motives are associated with a decreased likelihood of adhering to radical attitudes. Playing games for social reasons increases positive social outcomes (Reer and Krämer, 2019), while playing social games increases psychological wellbeing (Bowman et al., 2022). This could highlight profile 6 (the Socializer) as probably the “healthiest” motivational pattern, which of all profiles may still most likely inhibit the emergence of radical attitudes.

Although the study provides initial evidence in the relationship between gaming motivations, radical attitudes and well-known predictors of such attitudes, it is vital to acknowledge several limitations potentially affecting the interpretation of our findings: (1) This study should be regarded as a starting point contributing to the ongoing debate on the relationship between gaming and extremism, a discussion that frequently resurfaces in the aftermath of terrorist attacks. While our findings add empirical insights to a widely discussed issue, they do not establish causal links, highlighting the need for further research on the role of gaming in radicalization processes. (2) Self-reporting may introduce biases as participants' subjective assessments of their gaming motivations and radical attitudes could affect the reliability of the data collected. (3) Measurements regarding gaming motivations are limited, and only a selection of previously used items could be tested in the current study. This may especially be valid for the escapism/coping scale considering the recent argument that escapism could impose a dualistic structure associated with either positive or negative outcomes (Stenseng et al., 2021). (4) Another limitation concerns construct validity. In a study such as this, this can in principle be called into question by the difficulty of adequately operationalizing complex, multi-layered concepts such as game motivation and radical attitudes, which differ in their characteristics and can overlap in different individuals, potentially limiting the accuracy and completeness of our measurements. Finally, (5) the cross-sectional design of the study limits our ability to establish causal links or understand temporal dynamics between gaming motivations and radical attitudes over time. By situating gaming motives within broader extremism research, this study offers a novel perspective. But future work should further contextualize these findings within the wider landscape of terrorism studies and should incorporate additional game-related factors such as game content, genre, and time spent on gaming platforms to refine our understanding of potential risk and protective mechanisms.

Considering the specific game genres and their social environments could also provide deeper insights into how different gaming experiences relate to radical attitudes. Finally, our set of variables indicating radical thinking is also not exhaustive. While we align with decisive and particularly prevalent factors, multiple other types of radical or extremist attitudes and narratives are imaginable (Carter, 2018), such as misogyny or religious extremism. Future studies should complement possible effects motivational gaming profiles possibly have on e.g., nativism or the identification with the political fringe. While some effects reported in our structural equation model were statistically significant but small in size (e.g., β = 0.05), we interpret these cautiously. In line with recommendations by (Funder and Ozer 2019), such effects may still reflect meaningful patterns—especially in large samples and socially relevant domains—but are not assumed to have substantial individual-level impact.

To conclude, the current study explored profiles of gaming motivations (e.g., combinations of escapist, social, competitive, and hedonic motives) as proximal gaming-related susceptibility factors for radical outcomes, such as xenophobia, conspiracy beliefs, and the advocacy of political violence, including marginalization and anomie as mediators. Our work finds some weak relationships between gaming motivations and radical attitudes that are, when existent, mostly generated by experiences of marginalization and anomie. Profile 5, labeled “The Absorber”, characterized by multi-layered motivation, showed a weak direct inclination to promote measures of political violence, but also the strongest relationship to marginalization. In three other profiles, marginalization and anomie— as the main driving factor of radical outcomes—negatively mediated the relationship instead of further facilitating radical outcomes. Intriguingly, participants with a dominant escapism motive showed a negative direct relationship to radical outcomes such as xenophobia and conspiracy belief regarding COVID-19 and were largely unaffected by marginalization experiences. This is surprising, first because escapism and coping motives are associated with a variety of negative psychosocial outcomes (Wang et al., 2021; Tang et al., 2020). Second, gaming motivations are frequently negatively tied to radical outcomes, such as in players with a dominant social play motive. Thus, the motivational constitutions of players can contribute likewise negatively and positively to radical outcomes. However, the predictive quality of individual player motives seems on many occasions transmitted via external factors, thus limited and occasionally peculiar, suggesting that the exploration of proximal gaming related factors for radicalization requires a cautious and well-informed approach.

The study challenges monolithic views of gaming as a risk factor for radicalization and instead underscores its diverse psychological and social functions. While some gaming motives (e.g., escapism) might make players more vulnerable under specific conditions, others (e.g., social gaming, competitive gaming) appear to have protective effects. Rather than being a straightforward driver of extremism, gaming is better understood as a context-dependent space that can either buffer against or facilitate radicalization, depending on individual motivations and social context. Our analysis can be seen as an obstinate test that specifically searches for dark paths in the field of gaming. The conglomerate of effects however showed that protective affordances of gaming can, and usually do, outweigh its vulnerabilities.

## Data Availability

The dataset used in this study is not publicly available due to institutional and data protection agreements. Access to the dataset may be possible upon reasonable request, subject to approval by the responsible data holders and compliance with institutional and legal requirements. Requests to access the datasets should be directed to simon.greipl@ifkw.lmu.de. The survey data will be made publicly available at the University of Hamburg's research data repository, which is open and free to use, when all qualification theses/dissertations of the project staff have been completed.

## Footnotes

^1^We follow (Schmid 2013) considering radicalization as “a process whereby […] normal practices of dialogue, compromise and tolerance between political actors […] with diverging interests are abandoned by one or both sides in a conflict dyad in favour of a growing commitment to engage in confrontational tactics of conflict-waging” (p. 19). Radicalism and extremism are parts or states within this process, both rooted in discontent with and seek to fundamentally change the political system. However, while radicalism tends to be more open to rationality and pragmatic compromises, extremism rejects compromise and “manifests a closed mind and a distinct willingness to use violence against civilians” (Schmid, 2013, p. V). Extremists “tend to disregard the rule of law and reject pluralism in society” (Schmid, 2013, p. 9).
