# Supplementary material for: Tracking down the Candy Crush Terrorist: the fragile relation between gaming motives and radical attitudes
Source: Front Psychol. 2025 Oct 14;16:1585576. doi: 10.3389/fpsyg.2025.1585576 (PMC12558929; doi:10.3389/fpsyg.2025.1585576)
Supplement: Supplementary file 1 [file Table_1.docx]

Supplementary Table 1: Demographic Composition of Gamer Motivation Profiles: Sample Share, Age, and Gender

| **Profile** | **N** | **Overall sample share (%)** | **Age (Mean)** | **Male (%)** | **Female (%)** | **Diverse (%)** |
| --- | --- | --- | --- | --- | --- | --- |
| Escapist | 309 | 13.20 | 18.67 | 36.80 | 59.90 | 3.30 |
| Competitive-Escapist | 310 | 13.20 | 18.71 | 61.20 | 38.20 | 0.60 |
| Social-Escapist | 291 | 12.40 | 18.45 | 56.90 | 39.70 | 3.40 |
| Competitor | 328 | 14.00 | 18.48 | 80.10 | 18.30 | 1.50 |
| Absorber | 288 | 12.30 | 18.39 | 72.90 | 24.30 | 2.80 |
| Socializer | 212 | 9.10 | 18.64 | 68.40 | 30.70 | 0.90 |
| Recreationalist | 604 | 25.80 | 18.69 | 49.60 | 49.80 | 0.70 |

Supplementary Table 2: Demographics Composition of Gamer Motivation Profiles: Educational Background and Political Self-Positioning. Percentage distribution of respondents' highest educational qualification and mean scores for political self-positioning

| **Profile** | **Education** | | | | **Political self-positioning (Mean; 1= left, 10=right)** |
| --- | --- | --- | --- | --- | --- |
|  | **Lower secondary school (%)** | **Intermediate secondary school (%)** | **Higher secondary school diploma (%)** | **no qualification (%)** |  |
| Escapist | 1.60 | 23.60 | 73.80 | 1.00 | 4.04 |
| Competitive-Escapist | 3.60 | 20.10 | 75.00 | 1.30 | 4.29 |
| Social-Escapist | 5.20 | 24.50 | 69.70 | 0.70 | 4.09 |
| Competitor | 2.10 | 21.20 | 75.80 | 0.90 | 4.63 |
| Absorber | 4.50 | 28.10 | 64.90 | 2.40 | 4.26 |
| Socializer | 4.20 | 25.90 | 69.30 | 0.50 | 4.32 |
| Recreationalist | 2.00 | 15.90 | 81.10 | 1.00 | 4.33 |

Supplementary Table 3: Top 3 Reported Game Genres per Gamer Motivation Profile

| **Profile** | **Genre** | **N** | **%** |
| --- | --- | --- | --- |
| Escapist | Simulation and Sports | 92 | 29.80 |
|  | Puzzle And Party Games | 32 | 10.40 |
|  | Action-Adventure | 26 | 8.40 |
| Competitive-Escapist | Simulation and Sports | 85 | 27.40 |
|  | Multiplayer Online Battle Arena | 50 | 16.10 |
|  | Puzzle and Party Games | 32 | 10.30 |
| Social-Escapist | Simulation and Sports | 68 | 23.40 |
|  | Multiplayer Online Battle Arena | 53 | 18.20 |
|  | Action-Adventure | 22 | 7.60 |
| Competitor | Multiplayer Online Battle Arena | 71 | 21.60 |
|  | Simulation and Sports | 65 | 19.80 |
|  | Shooter | 36 | 11.00 |
| Absorber | Multiplayer Online Battle Arena | 89 | 30.90 |
|  | Simulation And Sports | 53 | 18.40 |
|  | Shooter | 34 | 11.80 |
| Socializer | Simulation and Sports | 50 | 23.60 |
|  | Multiplayer Online Battle Arena | 43 | 20.30 |
|  | Action-Adventure | 17 | 8.00 |
| Recreationalist | Simulation and Sports | 179 | 29.60 |
|  | Puzzle and Party Games | 82 | 13.60 |
|  | Multiplayer Online Battle Arena | 56 | 9.30 |

Supplementary Table 4: Descriptive Statistics (Mean; SD in brackets) of Radical Outcomes by Gamer Motivation Profile

| **Profile** | **Conspiracy COVID** | **Conspiracy Elite** | **Xenophobia** |  | **Acc. of Political Violence** |
| --- | --- | --- | --- | --- | --- |
| Escapist | 1.52 (0.74) | 1.94 (0.80) | 1.80 (0.75) |  | 1.39 (0.50) |
| Competitive-Escapist | 1.69 (0.85) | 2.11 (0.88) | 1.92 (0.79) |  | 1.48 (0.55) |
| Social-Escapist | 1.63 (0.83) | 1.98 (0.83) | 1.95 (0.74) |  | 1.50 (0.59) |
| Competitor | 1.53 (0.74) | 1.96 (0.84) | 1.91 (0.75) |  | 1.44 (0.52) |
| Absorber | 1.64 (0.80) | 2.10 (0.85) | 2.02 (0.79) |  | 1.56 (0.61) |
| Socializer | 1.44 (0.68) | 1.84 (0.78) | 1.88 (0.74) |  | 1.31 (0.45) |
| Recreationalist | 1.56 (0.77) | 1.86 (0.80) | 1.89 (0.73) |  | 1.35 (0.49) |
